# *Drosophila* olfaction: past, present and future

**DOI:** 10.1098/rspb.2022.2054

**Published:** 2022-12-21

**Authors:** Richard Benton

**Affiliations:** Center for Integrative Genomics, Faculty of Biology and Medicine, University of Lausanne, CH-1015 Lausanne, Switzerland

**Keywords:** *Drosophila melanogaster*, olfaction, olfactory receptor, neural circuit, neurophysiology, animal behaviour

## Abstract

Among the many wonders of nature, the sense of smell of the fly *Drosophila melanogaster* might seem, at first glance, of esoteric interest. Nevertheless, for over a century, the ‘nose’ of this insect has been an extraordinary system to explore questions in animal behaviour, ecology and evolution, neuroscience, physiology and molecular genetics. The insights gained are relevant for our understanding of the sensory biology of vertebrates, including humans, and other insect species, encompassing those detrimental to human health. Here, I present an overview of our current knowledge of *D. melanogaster* olfaction, from molecules to behaviours, with an emphasis on the historical motivations of studies and illustration of how technical innovations have enabled advances. I also highlight some of the pressing and long-term questions.

## 1. Introduction

Since they find their food with great certainty even in the dark, a habit that seemed to involve the sense of smell, I was led to take up an investigation of their reactions to odorous substances.

   Barrows, 1907 [1]

Originating in the work of William Barrows over a century ago [1], how *Drosophila melanogaster* detects and responds to odours has intrigued an ever-increasing number of researchers. Many early studies focused on analysing olfactory contributions to mating rituals [2] and the formation of olfactory memories [3], two of the three behaviours subject to pioneering neurogenetic dissection by Seymour Benzer and collaborators (as compellingly described in his biography *Time, love, memory* [4]). Molecular and cellular dissection of the structure and function of the *D. melanogaster* olfactory system progressed only modestly in the last decades of the twentieth century [5,6], especially by comparison with advances in our understanding of visual system development and photoreception [7,8]. However, work in other insect species—dating back to the 1870s when Jean-Henri Fabre ‘discovered’ chemical communication in moths [9]—yielded many insights into how animals represent odours in the brain to evoke behaviour. Several principles defined in these species were subsequently confirmed, refined and extended in *D. melanogaster* and vertebrates [10].

A watershed in insect olfactory research occurred in the late 1990s with the identification of *D. melanogaster* genes encoding olfactory receptors [11–13]. Similar to the impact of the earlier discovery of mammalian olfactory receptor genes [14], this breakthrough provided the foundation for investigating how odours are detected and olfactory circuit organization and function. *Drosophila melanogaster* olfaction now represents a model for sensory coding that is relevant for understanding similar processes in vertebrate brains but also continues to inform (and be informed by) studies in other insect species. Here I highlight—admittedly superficially and subjectively—some of the historical work and current knowledge of the *D. melanogaster* olfactory system, as well as open questions. I do not discuss in any detail the vast body of research on the olfactory systems of other species [15] (and little on the *D. melanogaster* larval olfactory system [16,17]), nor how olfactory signals integrate with other sensory information [18] or are represented in memories [19]. Rather, the goal is to present a brisk, but holistic, tour of how studying the fly's nose advances biological knowledge.

## 2. Receptors

**Figure 1.**
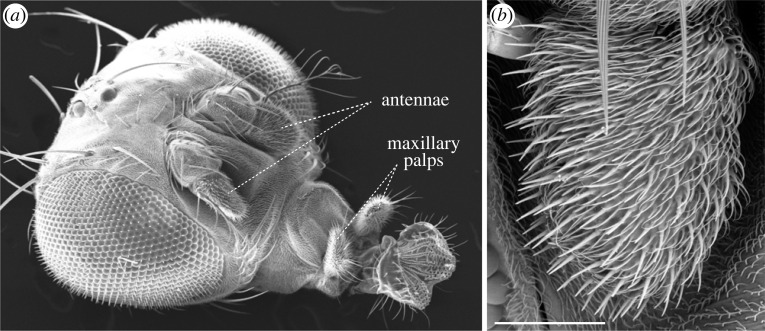
Olfactory organs. (*a*) Scanning electron micrograph (SEM) of the head of adult *D. melanogaster*, showing the two bilaterally symmetric olfactory organs. Adapted from [22] (copyright © Cold Spring Harbor Laboratory Press). (*b*) SEM of a *D. melanogaster* antenna, illustrating the dense array of morphologically diverse sensilla (which house olfactory sensory neuron dendrites) covering the surface. Scale bar, 50 µm. Adapted from [23].

Olfactory receptors convert chemical signals in the environment into electrical signals in the nose. In insects, there are two main families of olfactory receptors: odorant receptors (Ors) [11–13] and the more recently discovered ionotropic receptors (Irs) [20]. Early work revealed several similarities to the peripheral olfactory system of mammals: receptor families are generally large (encoded by dozens to thousands of genes per species) and have high sequence divergence, reflecting their role in detecting a vast number of different chemicals [21]. Moreover, in both insects and vertebrates, the majority of olfactory sensory neurons (OSNs) in the nose—the antenna and maxillary palp in insects (figure 1)—express just one receptor [24,25], which is the principal determinant of the sensitivity and breadth of odour recognition [26]. As described in the ‘Circuitry’ and ‘Function’ sections, knowledge of the receptors was also key to characterize structural and physiological properties of the peripheral olfactory system of *D. melanogaster*, which further illuminated parallels with mammals.

**Figure 2.**
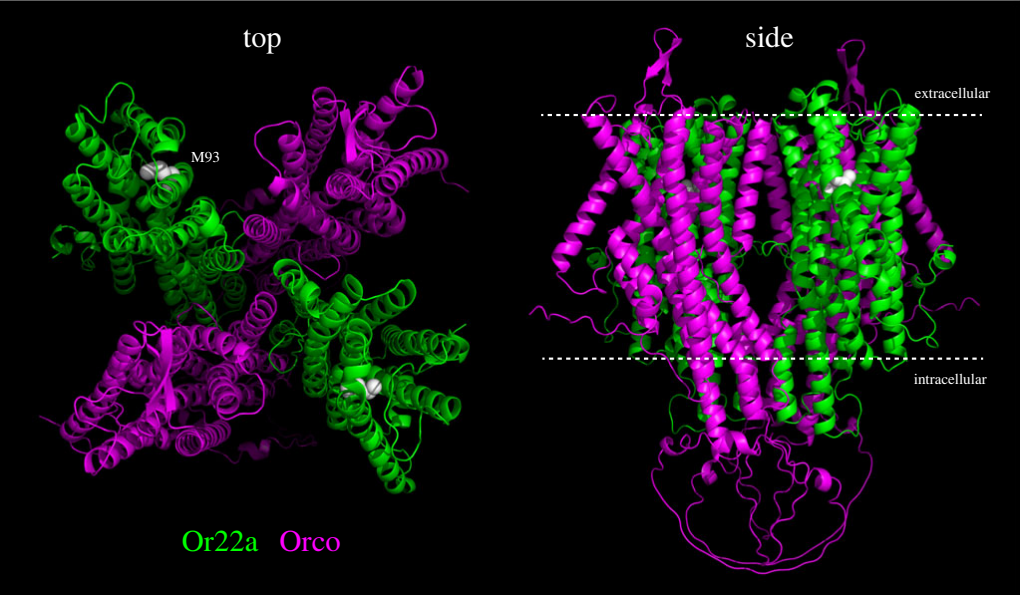
Olfactory receptors. Model of a hypothetical heterotetrameric complex of *D. melanogaster* Or22a and the co-receptor Orco (two subunits each). The approximate position of the plasma membrane is indicated in the side view. In Or22a subunits, the residue highlighted in white (M93) is a major contributor to defining behaviourally relevant odour response differences between *D. melanogaster* and *D. sechellia* Or22a orthologues [34]; this residue is located within the putative odour-binding site [35]. The ion channel pore is formed at the interface of all four subunits [36]. Models of protein monomers were predicted by AlphaFold2 [37,38]; these exhibit very strong similarity to cryo-electron microscopic (cryoEM) structures of Ors from other insects [35,36]. Models were aligned to the cryoEM structure of the Orco homotetramer from the fig wasp (*Apocrypta bakeri*) [36] using Coot [39] and visualized in PyMol v. 2.5.4. Although the stoichiometry of Or/Orco complexes is unknown, evidence suggests that they contain at least two tuning Or subunits [32,40].

The similarities extend only so far. Unlike vertebrate olfactory receptors, which belong to the G-protein coupled receptor (GPCR) superfamily [27], insect Ors and Irs function as odour-gated ion channels [28–30]. Both classes of channels are composed of subunits of a uniquely expressed ‘tuning’ receptor (which recognises odours) and one or more broadly expressed, family specific ‘co-receptors’ (e.g. Orco for Ors), which are essential for localization and signalling [30–33]. Ors are seven transmembrane domain proteins, a feature leading to long-held, incorrect assumptions that they were GPCRs. Rather, these proteins define a novel family of ion channels (figure 2), apparently absent in vertebrates, but with distant relatives (of unknown function) across invertebrates as well as in plants and unicellular eukaryotes [41–44]. Irs have evolved in protostomes from ionotropic glutamate receptors (iGluRs), a widely conserved family of ligand-gated ion channels best-known for their roles at synapses in the central nervous system [20,45]. Why insects use ionotropic transduction mechanisms to encode odours—contrasting with the reliance of vertebrates on metabotropic pathways—is unknown [46]. It might, for example, reflect a need for rapid signalling in OSNs in insects, which often encounter and navigate through spatially complex odour plumes in flight. But it might simply be a chance of evolution that different types of ancestral membrane receptors were adopted as the principal mechanisms of olfactory transduction in different lineages.

One outstanding question is how insect olfactory receptors interact with odour ligands and transduce this recognition into electrical signals. For Ors, advances were hampered by lack of similarity to known channels. A recent breakthrough came from the first cryo-electron microscopic structures of insect Ors—and indeed any animal olfactory receptor—that reveal important insights into how these proteins assemble into complexes and the mechanism by which odour binding gates the ion channel pore [35,36]. Of particular note are the first structural explanations for how an olfactory receptor can be activated by chemically diverse ligands [35]. Such structures—as well as remarkably similar *de novo* models of other receptors (figure 2) [42]—synergize with analysis of natural sequence variation and site-directed mutagenesis [34,35,47–51] to help define the molecular basis of receptor specificity and evolution. Irs may function similarly to their iGluR ancestors [30], and while no Ir structures are available, protein modelling in combination with mutational analyses have started to reveal principles of ligand-recognition and ion conduction of this family [30,52–54]. Beyond Ors and Irs, other types of proteins mediate peripheral sensation of ecologically important odours: CO_2_ is detected by two highly conserved members of the ‘Gustatory’ receptor (Gr) family (from which Ors evolved) [55,56], and an ammonium transporter is a sensor of ammonia [57].

**Figure 3.**
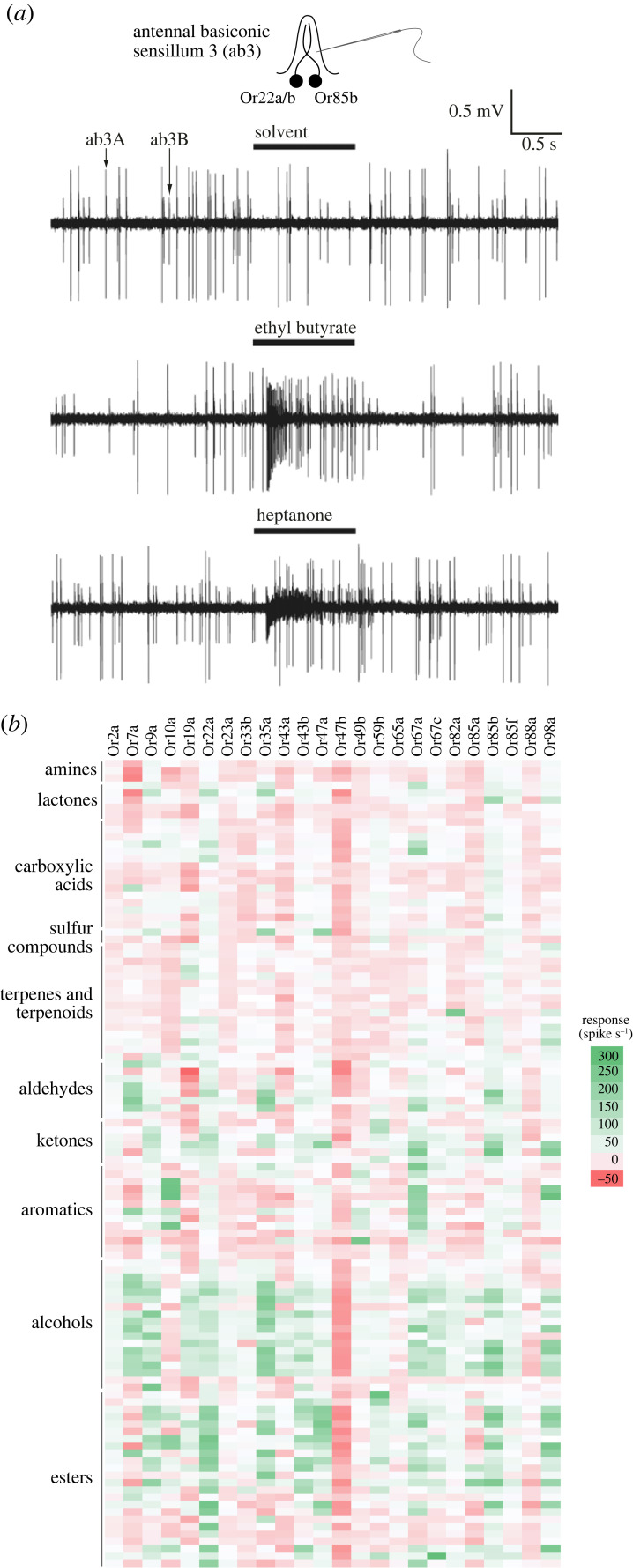
Olfactory function. (*a*) Electrophysiological recordings from the antennal basiconic sensillum 3 (ab3), which houses two neurons (ab3A and ab3B) that express Or22a/b and Or85b, respectively. The neurons can be distinguished both by ‘spike’ (action potential) amplitude and their responses to different odours (diluted to 0.001% v/v in the paraffin oil solvent and presented during 1 s). Adapted from [22] (copyright © Cold Spring Harbor Laboratory Press). (*b*) Combinatorial coding of odours by Ors: the first large-scale profiling of responses of many Ors to a chemically diverse panel of stimuli. Here, Ors were transgenically expressed in the ‘empty’ Or22a/b neuron (lacking the endogenous receptors) to provide a consistent cellular background for comparison of receptor function. Data are replotted from [61]; the scale is shown on the right. Negative responses reflect odours that decrease the basal spiking frequency of neurons. Some receptors for which no strong agonists were identified (e.g. Or47b) were later found to respond to pheromones [67,68].

*Drosophila melanogaster* has provided a facile experimental system for *in vivo* deorphanization of olfactory receptors. Odour-evoked activity can be easily measured by extracellular electrophysiological recordings of OSNs housed within porous sensory hairs (sensilla) on the surface of olfactory organs (figure 1) [22]. This ‘single sensillum recording’ method—initially developed in the mid-twentieth century for much larger insects [58,59]—has allowed identification of receptors/OSNs responding to, for example, food odours or pheromones [26,60–66] (figure 3*a*). Moreover, *D*. *melanogaster* mutants lacking specific tuning Ors or Irs have enabled use of the resultant ‘empty’ neurons as powerful *in vivo* heterologous expression systems for other receptors (including those from other insects) to determine their response profile [26,69–71] (figure 3*b*).

By contrast, *ex vivo* functional reconstitution of these receptors—in cultured mammalian or insect cells and frog oocytes—has been challenging: despite some successes [28–30,35,36,72,73], many Ors and Irs fail to exhibit odour-evoked current flow in other cell types. This limitation has constrained the aspirations of performing high-throughput ligand/receptor screening to identify novel (artificial) receptor agonists/antagonists, or perform large-scale, site-directed mutagenesis to define structure–activity relationships. The poor or absent activity of many olfactory receptors may be because such expression systems lack critical properties characteristic of these proteins' *in vivo* environment. Olfactory receptors localize to specialized sensory cilia of OSN dendrites that are bathed in an ion- and protein-rich lymph fluid within the sensillum [74,75]. Numerous perireceptor proteins (e.g. odorant binding proteins and odorant degrading enzymes) and accessory neuronal proteins (e.g. the CD36-related transporter Snmp1 or the ENaC-related channel Ppk25) contribute in diverse, often receptor-specific, ways to olfactory transduction [52,74,76–79]. Much remains to be discovered in the biochemistry and cell biology of how chemical cues are converted into electrical activity. Given the exceedingly small size of the sensory apparatus, many advances will depend upon technical innovations in cellular ultrastructural analysis [75,80].

## 3. Circuitry

**Figure 4.**
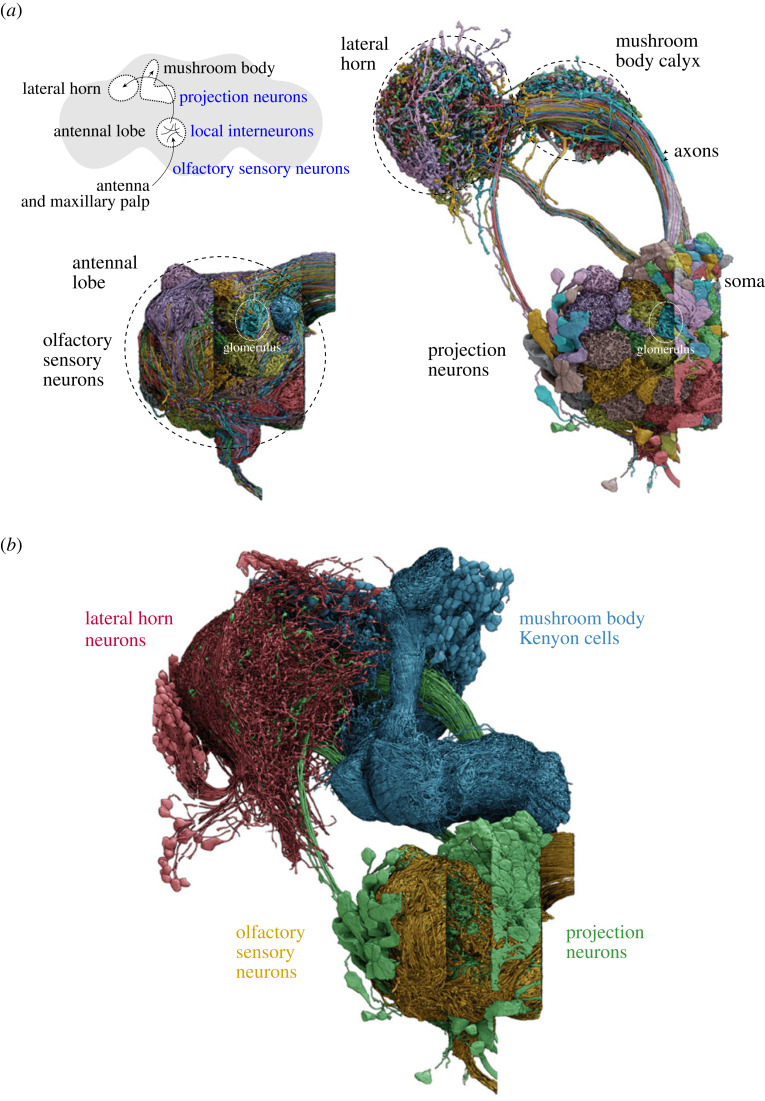
Olfactory circuits. (*a*) Top left: schematic of the principal paths of olfactory information flow in the *D. melanogaster* brain (outlined in grey), indicating the main neuron classes and brain regions. The circuitry is bilaterally symmetric (and most olfactory sensory neurons (OSNs) project to both antennal lobes) but only one hemibrain pathway is illustrated. Below are electron microscopic-resolution connectomic reconstructions of OSNs (including some antennal hygrosensory and thermosensory neurons [81–86]; contralateral innervations are cut off on the right side) and uniglomerular projection neurons (PNs) in the antennal lobe. Partner OSNs and PNs, converging on a common glomerulus, are colour-matched; one such glomerulus is highlighted in both images. For this PN class, a subset of the soma (located outside the lobe) are indicated with white arrowheads; black arrowheads point to the axons that project to the higher brain centres. (*b*) Connectomic reconstructions of the indicated neuronal populations. Data in (*a*,*b*) are adapted from [87], prepared by P. Schlegel; note that missing surface ‘strips’ in the middle of the antennal lobe are due to absent data.

The identification of olfactory receptor genes was also instrumental for neuroanatomical analysis of the *D. melanogaster* olfactory system. Receptor gene promoters form the basis of transgenic ‘drivers’ to visualize the innervations of the corresponding OSNs in the brain. A key principle is that OSNs expressing the same receptor project their axons to a common, discrete region of neuropil (a glomerulus) within the primary olfactory centre, the antennal lobe (figure 4*a*) [88,89]. This wiring pattern—originally observed in moths through dye back-filling of pheromone-sensing neurons [90]—is also a hallmark of the analogous olfactory bulb in vertebrates [91,92]. However, as OSN development in insects (see below) and in mammals [92] is very different, it is likely that glomerular segregation of OSNs is an evolutionary convergent, rather than conserved, property [15,93]. *Drosophila melanogaster* has excelled in olfactory circuit analysis because the combination of genetic tools and numerical simplicity (∼50 OSN classes) has enabled generation of an essentially complete receptor-to-glomerulus map [57,65,94–96]. This information synergizes powerfully with comprehensive knowledge of the odour-specificity of individual sensory channels (see ‘Function’ section) [26,61–66].

Within glomeruli, OSNs synapse with two types of interneurons: local interneurons (LNs) and projection neurons (PNs), whose anatomical properties were initially mostly studied using fortuitously identified enhancer-trap drivers and clonal labelling strategies [97–101]. More recently, electron microscopy-level analysis of the *D. melanogaster* brain [102] has prompted comprehensive synapse-resolution description of the olfactory system connectome [87,103–106]. LNs form connections between different glomeruli, exhibiting great diversity in the number and identity of glomeruli innervated, albeit with coarse-grained stereotypy [97]. Most PNs innervate single glomeruli (comprising an equivalent ∼50 classes as for OSNs) (figure 4*a*) and project their axons to higher brain centres: the lateral horn, which mediates innate responses to odours [107], and the mushroom body, a site of learned odour responses and sensory integration [19] (figure 4*a,b*). These centres are often considered insect analogues of the mammalian amygdala and piriform cortex, respectively.

The lateral horn does not exhibit the overt glomerular compartmentalization of the antennal lobe, but early work visualizing axons of single-labelled PNs and registering these images into a common ‘reference brain’, revealed spatially stereotyped PN innervations in this region [99–101] (figure 4*a*), concordant with its role in experience-independent odour-evoked behaviours. Further insights into how the antennal lobe glomerular map is transformed into the lateral horn neuronal map have required large-scale screening for sparsely expressed transgenic drivers [108,109], functional mapping of PN-lateral horn neuron connections using optogenetics [110], together with connectomics. These studies suggest the existence of a large number (greater than 250) of lateral horn neuron types [108]. Some of these neurons display only local innervations, while ‘output neurons’—which receive strong input from a stereotyped set of 1–10 PN classes [108]—project to other brain regions [108,110]. The unanticipated number and diversity of lateral horn neurons is likely to reflect the numerous (but largely unknown) ways in which olfactory inputs from PNs are integrated, processed and segregated to downstream circuits [107]. Lateral horn output neurons do not project directly to the ventral nerve cord—the insect analogue of the spinal cord—implying the existence of additional (though perhaps not many [111] and preprint [112]) layers of circuitry, before reaching motoneurons.

Within the mushroom body, PNs synapse with Kenyon cells (figure 4*b*), with up to ∼10 PNs converging onto individual mushroom body neurons. An important question is the degree to which PN connectivity in this brain region is stereotyped (as in the lateral horn) or whether random combinations of PN types synapse on common Kenyon cells. The latter scenario has been suggested to provide an anatomical basis to enhance odour discrimination and/or a template to permit ‘meaning’ to be imparted onto unpredictable olfactory stimuli through learning [104,113,114]. Initial low-resolution maps of PN innervations suggested the existence of a degree of zonal organization in the mushroom body [99,115,116], while subsequent single cell-resolution surveys of PN–Kenyon cell connectivity [114] (and related functional studies [117]) in a subset of the circuitry provided evidence that Kenyon cells receive input from random sets of PNs. Recent comprehensive analysis of the PN–Kenyon cell connectome revealed a more nuanced situation, where there is a degree of non-random structure [104]. For example, PNs transmitting food-related odour signals converge onto Kenyon cells at frequencies above those expected by chance.

Beyond these few examples, our understanding of olfactory circuit organization has been revolutionized by connectomics, which is uncovering many other features of known circuit elements (e.g. axo-axonic connections between PNs [103]) and new neuron types (e.g. those directly linking the lateral horn and mushroom body [118,119]). Such high-resolution anatomical data presents both opportunities and challenges [120]: although we can now trace sensory pathways from OSNs to deep within the brain, the wide dispersal of information across the network (e.g. preprints [112,121]) is daunting, as it remains non-trivial to investigate the physiological and behavioural relevance of such complex connectivity.

## 4. Development

Visualization of individual olfactory circuit elements reinvigorated historical interest (e.g. [122,123]) in olfactory system development, notably the specification and wiring of OSNs and PNs (study of LNs is still in its infancy [124,125]). The reproducible one-to-one matching in the antennal lobe of the ∼50 distinct classes of OSNs and PNs that are born at different times, in different places and in distinct ways during development is an incredible example of biological precision. These neuron types exemplify two emblematic strategies of insect nervous system development: OSNs derive from short lineages of sensory organ precursors (SOPs) in the late larval/early pupal antennal imaginal disc, while PNs are produced by a long series of asymmetric divisions of neuroblasts (stem cells) in the central brain during embryonic and larval stages [126–128]. Studies of how these neurons acquire their identity and wire up correctly both inform, and are informed by, knowledge of developmental algorithms and neural guidance molecules of many regions of the nervous system in *D. melanogaster* and other animals.

Thanks to a combination of genetic perturbations and spatio-temporally precise cell-labelling methods [126–129], we now have a good (albeit incomplete) understanding of OSN and PN development. For OSNs, a fate map of SOPs in the antennal disc has been defined: each SOP gives rise to a particular morphological type of sensillum (basiconic, intermediate, trichoid, coeloconic) housing a stereotyped combination of 1–4 OSNs [123,126,127,130–134]. How this map is established is unclear, but likely relies upon conserved disc patterning factors such as Wingless and Hedgehog [126,135]. OSN fate emerges through the action of a hierarchy of transcription factors—from ‘proneural’ factors demarcating the Or and Ir olfactory subsystems, to those defining sensillum type or individual OSN classes—and Notch signalling-dependent asymmetric cell divisions [133,134]. The identity of a given OSN class is realised by the expression of a specific olfactory receptor gene [25,136–139] and a (presumably unique) set of axon guidance molecules [140,141]. Within PN lineages, distinct PN classes are born in a highly stereotyped order during embryonic and larval development [98,142]. This birth order-dependent patterning relies, in part, upon a temporal gradient of a transcription factor (Chinmo) [143,144]. This protein is thought to regulate the expression of PN class-specific combinations of guidance molecules that control dendritic wiring in the antennal lobe and/or axonal projections in the higher olfactory centres [128,145,146].

How do the diverse types of OSNs and PNs assemble together to form the stereotyped glomerular structure of the antennal lobe? Several general principles are known [128,145,146]. First, PN dendrites enter the lobe first where they establish an initial map of protoglomeruli [147], using both long-range spatial information (e.g. a gradient of Semaphorin-1a [148–151]) and local cues (e.g. Capricious, which has heterogeneous glomerular expression [152]). Second, OSN axons from the antenna bifurcate into distinct tracts (requiring Semaphorin-2b [153]) before entering the antennal lobe, where they coarsely target the right region through cellular interactions with other OSNs and glia mediated by a multitude of neural guidance/adhesion molecules (e.g. Robos, Dscam, N-cadherin [154–157]). Third, OSN axons and PN dendrites—now positioned closely together in the lobe—wire up with high specificity using ‘match-maker’ molecules, which are expressed in partner OSNs and PNs (e.g. Teneurins [158]). Finally, OSN-PN synapses mature to render these connections functional (again using Teneurins [159]). Despite this framework, the wiring problem remains fantastically complex and still only superficially understood at the molecular and cellular levels.

Three technological advances promise to further enhance our understanding of olfactory circuit development. First, time-lapse imaging of the developing antennal lobe—using lattice light-sheet microscopy of antennal-brain explants—has permitted observation of OSN targeting behaviour that was previously interpreted only from snapshots of fixed tissue [160]. Second, ‘omics’ technologies, notably single-cell/nuclear RNA sequencing of OSNs and PNs [140,141,161–164] has revealed comprehensive lists of the molecular differences between populations that might explain their distinct properties both during development and in adults. Of course, determining the functional significance (if any) of differentially expressed genes requires substantial investment (e.g. [140,163]). Finally, proteomic profiling of cell-surface molecules in PNs has been particularly fruitful in identifying novel regulators of dendritic wiring [165]. This approach has also helped to make a causal link between a transcription factor (Acj6) defining neuron fate, the set of cell-surface proteins regulated by this transcription factor, and dendritic wiring specificity [166]. A goal of understanding olfactory circuit assembly—to decrypt and eventually reprogramme the successive combinatorial codes of transcription factors and their cellular executors that define wiring specificity—seems within reach.

## 5. Function

The anatomical map of olfactory circuitry provides a static picture of how olfactory information might be transmitted from the antenna to the brain. But understanding how odours are encoded as neuronal activity patterns requires physiological measurements and manipulations throughout this network [167,168]. Key initial insights into coding mechanisms came from surveys of odour-evoked activity in OSNs. These screens have mostly been via electrophysiological recordings in olfactory sensilla [26,60–66] (figure 3), but complemented by imaging OSN activity in their axon termini in the antennal lobe using genetically encoded sensors (e.g. the calcium reporter GCaMP) [169,170]. In the latter approach, the stereotypical organization of the lobe allows glomerular activity to be related to the corresponding receptors.

These efforts revealed a number of important observations that extended principles of odour coding originally proposed in other insects (notably, honeybees [171,172]) and mice [173]. First, olfactory receptor/OSN response profiles can vary from very narrow (such as those detecting a specific pheromone) to very broad (such as those detecting food-derived volatiles). Second, most individual odours activate multiple classes of OSNs. Third, the detection threshold of OSNs that respond to the same chemical can vary over several orders of magnitude. Fourth, OSNs exhibit a basal firing rate (potentially due to spontaneous receptor channel gating [29]) and many odours lead to a reduction in this activity [61]; basal firing can also be decreased indirectly through non-synaptic electrical interactions (ephaptic coupling) between OSNs housed in the same sensillum [174,175]. Lastly, there is substantial diversity in the temporal dynamics of odour responses, including onset latency upon stimulus presentation or persistence of firing after odour removal [26,176,177]. These properties have led to a model in which individual odours are represented as a ‘combinatorial code’ of OSN activity, whose spatio-temporal patterns of activation (and possibly inhibition) can inform the brain of the identity and intensity of a stimulus. This model, applicable to both insects and vertebrates, is compelling, not least for its ability to explain how olfactory systems might discriminate many more stimuli than there are sensory receptors. Surprisingly, it still remains unclear whether this model is valid for the encoding of the identity of individual odours. *D. melanogaster* responds behaviourally to almost every odour presented to it (e.g. [178]), but we do not know how many stimuli this species can discriminate. Explicit experimental tests of combinatorial coding have been rather limited (e.g. [179], or in larvae [180]). Moreover, there are now many examples of OSN classes that exhibit unique, narrow tuning to chemicals of particular ecological relevance (see ‘Ecology’ section). This species might therefore rely substantially on ‘labelled line’ olfactory coding, in which individual odours trigger specific behaviours through dedicated neural pathways [181,182]. Of course, natural odour sources (e.g. fermenting fruit) emit complex chemical blends, for which combinatorial neural representations are inherent to stimulus processing.

How the rich sensory information content that reaches the antennal lobe is transformed from OSNs to PNs has been subject to intensive investigations, through optical imaging at both of these neuronal layers, and electrophysiological recordings from PNs innervating specific glomeruli [168–170,183–185]. While OSNs provide the main excitatory drive to the PNs with which they directly synapse, the relationship between OSN and PN firing rates is nonlinear. In part, this relationship reflects the nature of OSN:PN synapses: the pooling of several-fold more OSNs than PNs in most glomeruli enables high sensitivity of PNs to low OSN activity (i.e. low odour concentrations) and highly reliable activation of PNs [186,187]. But transformation of signals from OSNs to PNs is also influenced by interactions between glomeruli. The most prominent of these is lateral inhibition, where strong OSN activity can suppress firing in other OSN populations via GABAergic local interneurons [188–190]. Inter-glomerular interactions can be global across the lobe but can also occur between certain combinations of glomeruli. Such inhibition is likely to underlie enhancement of the signal-to-noise ratio of glomerular odour representations, ensuring that strong signals through one pair of OSN/PN classes are not ‘muddied’ by weaker activation of others. Lateral excitation has also been reported, evident as responses of PNs to odours in the absence of activity in their partner OSNs [191,192], but the significance of this phenomenon is less clear.

The antennal lobe is also an important site of neuromodulation, influenced by the internal state of the animal. For example, when flies are starved, odour responses in a glomerulus detecting attractive food odours are increased, due to the integrated signalling of insulin and short neuropeptide F (a homologue of vertebrate NPY), which elevates calcium levels in OSN presynaptic termini [193]. Conversely, the responsiveness of a glomerulus detecting aversive odours is decreased through the action of a Tachykinin (a homologue of vertebrate Substance P) [194]. Many neuropeptides (or their receptors) have been detected in the antennal lobe [194–196], often at heterogeneous levels across glomeruli, suggesting that odour representation in this primary olfactory centre can be altered in a circuit-specific manner in response to many different external influences (e.g. mating, feeding and health status).

Studies of odour representations in higher olfactory centres are starting to reveal how these brain regions might direct an animal's behaviours. In the lateral horn, population-level calcium imaging, single-cell level electrophysiology and connectomics data suggest that odour-evoked activity in different PN classes is integrated by third-order neurons and categorized according to hedonic value (e.g. ‘attractive’ or ‘aversive’) [107,108,110,197]. While this framework is useful for exploration of how this centre directs innate odour-evoked behaviour, it is certainly simplistic, belying the anatomical complexity of this brain region (see the ‘Circuitry’ section above). An ‘attractive’ stimulus might manifest in many different behaviours (e.g. approach to the source, feeding, social aggregation or oviposition). Furthermore, an odour might be attractive or aversive to a fly depending upon the presence of other environmental cues or the animal's internal state (e.g. [198,199]). More generally, study of PN-to-lateral horn neuron neurotransmission has been a useful model to assess the functional significance of anatomical features revealed by the connectome, including synapse density and distance from the soma [200]; these insights should be pertinent throughout the nervous system.

Odour representations in the mushroom body of *D. melanogaster* (and other insects) are rather different to those in the lateral horn. Initial studies found that, in contrast to PNs, Kenyon cells display extremely sparse responses to odour stimulation, likely due to their high firing threshold and (at-the-time) presumed random connectivity with PNs [201–203]. Such properties might be advantageous for high capacity, non-overlapping neuronal representations of different stimuli in a centre where associative learning occurs. Recent volumetric calcium imaging has permitted a more comprehensive view of odour-induced activity in Kenyon cells [204]. Interestingly, this work has revealed greater spatial organization than previously appreciated—potentially matching the degree of non-random wiring indicated by connectomics [104]—as well as the existence of cells that respond to mixtures of odours, but not their individual components [204].

While global analyses of odour representations in the higher brain centres is clearly an exciting work-in-progress, focused attention over the past 15 years on the olfactory pathway detecting the sex pheromone *cis*-vaccenyl acetate is helping to relate its structural and functional properties to behaviour. This male-specific pheromone has multiple roles in *D. melanogaster* [205], notably its dual action in suppressing male courtship (to avoid homosexual advances) and promotion of female receptivity [206]. Unlike striking peripheral sexual dimorphisms in pheromone pathways in other insects [10], OSNs detecting *cis*-vaccenyl acetate (expressing Or67d) and the downstream PNs both display similar pheromone responsiveness in males and females [206–208]. Such similarity begs the question of how this pheromone evokes different behaviours in males and females. This pathway forms part of the so-called ‘Fruitless’ circuitry, which comprises sensory, inter- and moto- neurons that control courtship [209,210]. Using genetic drivers for this circuitry, it has been possible to anatomically and functionally characterize the *cis*-vaccenyl acetate pathway from the lateral horn through the ‘P1’ brain centre, which controls several sexually dimorphic behaviours, to descending neurons innervating the ventral nerve cord [111,211,212]. Importantly, these studies revealed sex-specific wiring between PNs and lateral horn neurons—offering a cellular explanation for the sex-specific behavioural responses to *cis*-vaccenyl acetate [212]—as well as other important insights into sensory coding, integration and plasticity [111,211,213] and preprint [121].

## 6. Behaviour

**Figure 5.**
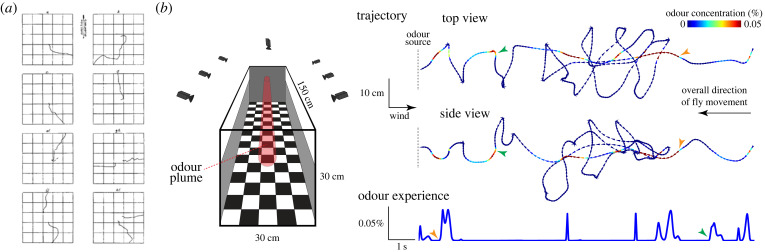
Olfactory behaviour. (*a*) Historical tracking: manually traced paths of individual walking *D. ampelophila* (the former name for *D. melanogaster*) from the edge of a 5 × 5 inch arena toward a piece of fermenting banana at the centre. Reproduced from [1], with permission. (*b*) State-of-the-art tracking: schematic of a wind tunnel through which a controlled odour plume can be introduced; a multi-camera system enables automated three-dimensional tracking of flies interacting with this plume in flight. On the right is the trajectory (top and side views) of *D. melanogaster* in a continuous plume of ethanol. Grey dashed lines on the left indicate the upwind wall of the tunnel towards which the animal flies. The trajectories are colour-coded for the instantaneous concentration of odour at a given point in the plume (as measured using a photoionization detector); the reconstructed odour experience of the animal over the course of its flight is plotted below the trajectories. Two time-synchronized reference points are indicated with orange and green arrowheads. Adapted from [214], with permission.

The *raison d’être* of the olfactory system is, of course, to tell the fly how to react when it encounters an odour. Typically, olfactory behaviours are assessed using simple assays in which flies can move toward or away from a chemical source, from which inferences about the ‘attractiveness’ or ‘aversiveness’ of an odour are made (figure 5*a*). Flies might also be offered a choice of two or more odours (or odour concentrations), to determine how well animals discriminate and/or value different stimuli [215]. Technological advancements in automated animal tracking [216–218], as well as more sophisticated methods for odour delivery and measurement [219–223], have enhanced the throughput, sensitivity and analytical objectivity of assays, as well as enabling study of navigation within odour gradients and plumes when walking or during flight [214,220,222,224,225] (figure 5*b*). Perhaps unsurprisingly, complex natural blends (such as vinegar or fruits) generally evoke the strongest behavioural responses. Consequently, to relate behaviours to specific receptor genes and circuits, there has been much interest in the innate actions, such as courtship or aggression, induced by single volatile pheromones (including *cis*-vaccenyl acetate) [67,68,206,210,226], as well as responses to other environmental chemicals that specifically activate single OSN populations (e.g. [57,227–230]).

Many odour-evoked behaviours have been causally linked to specific olfactory pathways, typically through analysis of olfactory receptor mutants or animals in which specific OSN populations are ablated or artificially activated. The availability of driver lines for nearly all OSN populations has enabled systematic screening for behaviours evoked upon activation of individual pathways [179,231,232]. However, robust responses are rarely observed, indicating that meaningful perception of olfactory stimuli in the brain might require combinatorial activation of multiple sensory pathways with appropriate intensity and temporal precision. In this light, it is unsurprising that, deeper in the brain, olfactory behaviours have still mostly only been correlated with physiological representations (e.g. activation of putative ‘positive’ or ‘negative’ valence regions in the lateral horn [233] or sexually dimorphic pheromone responses [212]). However, some causal evidence has been obtained when selective driver lines permit targeted perturbation of higher order circuit elements (e.g. [197,234] and preprints [112,121]).

Interpreting behavioural responses can be tricky: subtle differences in culture conditions, genetic background and the assay itself can influence how animals behave, potentially masking effects of the desired experimental intervention. While frequently frustrating for researchers, behavioural variability is now being explored as an inherently interesting phenomenon, through examination of the individuality of olfactory responses of genetically identical individuals [235]. Conceptually, inter-individual variation in olfactory behaviour could be due to structural and functional differences at many levels of the olfactory circuitry [236]. Although the *D. melanogaster* olfactory system is considered to be largely genetically hard-wired, differences between isogenic animals [75,97,106] may result from imprecision in developmental processes [237] and/or fluctuations in the internal state of the animal impacted by a plethora of factors (e.g. feeding status, infection and prior sexual or social interactions). A recent preprint indicates that the sensory periphery is at least one site underlying inter-individual behavioural variation [238].

There is also growing appreciation that, as in humans, odours induce many other responses in *D. melanogaster* that do not directly involve locomotor circuitry, but rather impinge upon other neural or endocrine pathways. For example, olfactory perception (or lack of) can affect taste sensation (preprint [239]), modulate food ingestion and metabolism [240], contribute to maintenance and development of blood progenitors [241,242], as well as influence general stress responses and lifespan [243]. When flies do not visibly react to an odour, the olfactory system might still be meaningfully processing the sensory information.

## 7. Ecology

To keep pace with the progress in our understanding of *D. melanogaster*'s olfactory neurobiology, increasing efforts aim to better appreciate the ecology of this species. Such knowledge is likely to help identify relevant ligands for sensory receptors and to describe naturalistic odour-evoked behaviours. It is unclear why entomologist Charles Woodworth chose to culture *D. melanogaster* at the beginning of the twentieth century [244], but the flourishing of the species in the laboratory was a natural extension of its cosmopolitan distribution and commensal relationship with humans [245]. The presence of *D. melanogaster* in orchards, wineries, household compost bins and fruit bowls attests to its ‘generalist’ ecology, capable of feeding and breeding on a wide range of fermenting vegetal substrates. Consequently, many early olfactory studies screened for physiologically and/or behaviourally active odour ligands among individual, off-the-shelf, synthetic chemicals found in the complex bouquet of ripe, over-ripe and rotting fruits as well as the microbial catalysts (notably yeasts) of fermentation [246].

More recently, the odorous world of *D. melanogaster* has been further probed by analysing volatiles produced by pathogenic bacteria [230] and fungi [247], predators (notably parasitoid wasps) [248], as well as the identification of pheromones controlling sexual and other social behaviours [67,68,249]. Adopting a fly's perspective of the chemical world has helped to discover potent ligands for previously ‘orphan’ receptors.

By contrast, there has still been very limited examination of the olfactory behaviours of *D. melanogaster* in nature. This may be understandable, given the challenges of interpreting odour-dependent behaviours even under well-controlled laboratory assays. However, the few field studies of *D. melanogaster* have proven to be illuminating, including description of social interactions of flies near fruits [250,251] or the discovery of a deceptive pollination system of the Solomon arum lily, which produces a remarkably faithful odour mimicry of fermentation to attract *D. melanogaster* as an unwitting courier for its pollen [252]. One ambitious investigation located a potential origin and ancestral chemical ecology of wild *D. melanogaster*—not associated with contemporary human settlements—within the balsam tree forests of Zimbabwe [253]. Here the wild flies appeared to exhibit seasonal specialism upon marula fruit (*Sclerocarya birrea*), an important food source for historical tribes of this region, leading to a plausible model for the transition of this species to human commensalism and the extant generalist ecology [253].

## 8. Evolution

Study of the development and function of *D. melanogaster*'s olfactory circuits has benefited from their (mostly) stereotyped properties, enabling observation of reproducible phenotypes (both wild-type and mutant) across individuals. However, olfactory systems are highly dynamic over evolutionary timescales, shaped in part by changing environmental selection pressures, such as novel food odour signals when individuals colonize a new ecological niche. Given the enormous number of species and habitat diversity of insects, their olfactory systems have long been interesting models to explore the genetic, cellular and physiological basis of nervous system and behavioural evolution [129,254–257].

The deep foundation of knowledge of molecular, anatomical and physiological properties of the *D. melanogaster* olfactory system has made this a useful ‘anchor’ species for evolutionary comparisons at a range of evolutionary divergence times. Analysis of microevolution between geographically separated *D. melanogaster* strains, which may have diverged only a few thousand years ago has revealed that olfactory receptors (and other chemosensory protein families) display some of the strongest signals of recent selection, highlighting their potential roles as genetic ‘first-responders’ to new environments [47]. However, successful association of intraspecific genetic variation to physiological or behavioural differences has only rarely been achieved [49,258–260].

Macroevolutionary differences, across closely or more-distantly related drosophilid species, are widely documented, with particular interest in comparisons of the cosmopolitan generalist *D. melanogaster* with specialist species, such as the island endemic *Drosophila sechellia*, which feeds and breeds exclusively on the ‘noni’ fruit of the *Morinda citrifolia* shrub [34,54,70,257,261,262], the invasive agricultural pest *Drosophila suzukii*, which has evolved preference for ripe, rather than fermenting, fruit [263–267], or the herbivorous drosophilid *Scaptomyza flava* [268]. Together with comparative studies in many other insects, this work has revealed numerous examples of changes in odour response properties, reflecting in most cases evolution of olfactory receptors. In some cases, the causal molecular basis of functional changes in receptors has been mapped, demonstrating that while single amino acid substitutions can substantially modify odour responses (figure 2), species-specific tuning properties typically depend on multiple sites within (and beyond) the presumed ligand-binding site (e.g. [34,48,53,54]).

A second type of species-specific neuronal change is the increase (or decrease) in OSN population size, with commensurate changes in glomerular volume [34,54,262,269–271]. While expanded OSN classes are frequently those that detect ecologically important stimuli for a species, neither the developmental basis nor the functional significance of these presumed evolutionary adaptations are known. The non-pleiotropic functions of olfactory receptors (and the OSNs in which they are expressed) might explain why most described examples of evolutionary variation are within the sensory periphery. However, this bias probably largely reflects the experimental accessibility of this part of the olfactory system. We know very little about if and how other olfactory circuit elements have evolved, although subtle anatomical differences between species in PN innervations of higher brain centres have been observed [34]. Comparative studies of (contact) chemosensory pathways between drosophilids illustrate the potential for functional diversification of central circuit elements to explain species-specific behaviours [272].

The evolution of completely new olfactory circuits within the drosophilid phylogeny appears rare. One example exists in a class of pheromone-sensing sensilla, which houses a single neuron in the majority of drosophilids (the Or67d *cis*-vaccenyl acetate sensor) but a second, distinct neuron in a subset of species, including *Drosophila mojavensis* [273–275]. How new neurons appear during evolution is unclear, although work in *D. melanogaster* suggests that changes in the precise developmental patterning of programmed cell death—which removes many of the potential neurons in the olfactory sensory lineages—might be sufficient to create new OSN classes [273].

These studies highlight the core challenge of comparative neurobiology in balancing phylogenetic proximity and phenotypic divergence. Comparisons of closely related species facilitate identification of differences in traits and their experimental characterization, especially if these species produce fertile hybrids for genetic mapping, or genetic tools can be easily exported from *D. melanogaster* [34,257,272,276]. However, more dramatic changes in neuronal circuit structure and function may only be seen between species where it is harder to go beyond purely descriptive analysis. Of course, it is not always necessary (and probably often impossible) to determine the causal genetic basis of species differences. There is much current interest in harnessing genome-editing methods (notably CRISPR/Cas9) for molecular genetic characterization of the olfactory systems of both historically important species (e.g. moths [277–279]) and newer model systems (e.g. mosquitoes [280,281] and ants [282,283]). These studies can illuminate interesting properties of insect olfactory systems distinct from those of *D. melanogaster*, without necessarily providing a mechanistic basis for the evolutionary differences.

## 9. Applications

Since the discovery of olfactory receptors in *D. melanogaster*, the potential for application of this knowledge to combat the devastating impact of insect disease vectors (such as mosquitoes and tsetse flies) and agricultural pests (such as corn rootworm and locusts) has been widely recognized [284,285]. These harmful species depend heavily on their sense of smell for host animal- or host plant-seeking as well for reproductive and other social behaviours. It has been logically reasoned that characterization of peripheral olfactory receptors (and perireceptor proteins) in such species—following the lead in the benign *D. melanogaster*—could offer targets for pharmacological intervention to interfere with these odour-guided behaviours.

Soberingly, this ‘reverse chemical ecology’ approach has seen only limited progress. Technological advancements in sequencing have enabled identification of olfactory receptors in an enormous number of pest insect species [286,287]. However, only a minute fraction of these receptors has been functionally characterized, sometimes using *D. melanogaster* OSNs as a heterologous expression system [71,286–289]. Moreover, from information within at least the public domain, very few receptors have been subject to screens for artificial agonists or antagonists (e.g. [290–293]), although complementary chemoinformatic approaches to identify novel chemical modulators of receptors *in silico* show promise [294–296]. Some of these chemicals induce behaviours in laboratory-based assays [291–294,297], but there is little evidence of successful field trials.

Akin to the many ways in which drug development pipelines can fail, olfactory receptor-based efforts to identify new insect behavioural control molecules face numerous challenges. These include target specificity (many studies focused on (ant)agonists of the Or co-receptor Orco [290,294,297], which would likely affect all insect species), volatility, stability, non-toxicity to humans and cost of production. It remains to be seen whether reverse chemical ecology will ever yield more promising solutions than traditional, highly successful strategies of olfaction-based integrated pest management, which use natural semiochemicals with potent behavioural influence (e.g. pheromones) as the basis of lures or repellents [298,299]. Here, molecular knowledge of how a species detects a chemical cue is ultimately unnecessary.

A second potential avenue for practical application of insect olfactory receptors is in artificial chemosensors, with diverse applications, for example, in medical diagnostics, food quality assessment and environmental monitoring [73]. Theoretically, these proteins are ideal for this purpose: across millions of insect species there could be tens of millions of receptors with distinct chemical specificity and, in principle, they act autonomously as ligand-gated ion channels. Practically, however, progress has been slow, constrained by limitations in the reliability of heterologous functional expression of receptors. Moreover, despite some pioneering bioengineering efforts (e.g. [300]) integrating biological sensors into sensitive and robust optical- or electrical-based recording devices remains a huge technical challenge.

## 10. Perspectives

Barrows’ 1907 study [1] is remarkable for its foundational observations of *D. melanogaster* olfaction: the selectivity of odour responses and synergistic effect of odour mixtures, the rudiments of chemotaxis behaviour (in flying and walking animals), the importance of an animal's internal state, and the identification of the key sensory organs. Since that work, there have been enormous advances in our understanding of all of these—and many other—aspects of olfaction in this species. Yet, as illustrated by the open issues highlighted in this review (box 1), we cannot claim to be able to explain how the olfactory system is built or functions in much more than a partial way. Like many seemingly simple phenomena, the sense of smell of the fly is an extraordinarily complex problem, requiring integration of knowledge of genetics and molecular biology, cell and developmental biology, neuroscience and physiology, ecology and evolution. But this complexity offers the benefit that new insights have far-reaching significance in biology, from animal behaviour, neural circuit construction, ion channel function and gene regulation, to name just a few areas. More generally, during a research era where powerful new technologies, such as genome engineering and single-cell transcriptomics, open the doors to new questions and new model systems, the nose of *D. melanogaster* provides a useful reminder of the equal merits of focus. The novel, often surprising, scientific insights gained from study of the sense of smell of the fly, emerged because new results—whether a puzzling observation or a large dataset—could be better interpreted in a system where we already have a rich intellectual framework.

Box 1. **Five areas of specific and open questions in *D. melanogaster* olfaction**

1. How do olfactory receptors recognize odours with narrow or broad specificity? How do they collaborate with perireceptor proteins and sensillar structural properties to mediate sensitive and dynamic odour detection? What is the mechanistic basis by which sensory response profiles change over evolutionary timescales?
2. What are the complete developmental pathways specifying the diversity in neuronal fate and wiring properties at different layers of the circuitry? How are synaptic partners matched up with precision? What are the molecular and cellular mechanisms underlying the evolution of modified—or completely new—olfactory pathways?
3. How important is the combinatorial code for determining odour identity? What is the role of temporal properties of neuronal activity in odour coding? What are the physiologically important anatomical connections within the olfactory connectome? What is the logic of olfactory circuit organization underlying innate versus learned odour-driven responses?
4. What are the natural pertinent odour signals in *D. melanogaster*'s ecological niche? How are olfactory behaviours observed in the laboratory related to those in nature? How are olfactory cues integrated with other sensory information to drive complex behaviours, such as odour plume navigation? How and why are olfactory behaviours variable both between genetically identical individuals, and within an individual's lifetime?
5. (How) can the information from studies of *D. melanogaster* be usefully exploited to control odour-driven behaviours of insect vectors of disease and agricultural pests, or in engineering artificial biosensors?

## Data Availability

This article has no additional data.
